# The Evolutionary Biology of Musical Rhythm: Was Darwin Wrong?

**DOI:** 10.1371/journal.pbio.1001821

**Published:** 2014-03-25

**Authors:** Aniruddh D. Patel

**Affiliations:** Department of Psychology, Tufts University, Medford, Massachusetts, United States of America; McGill University, Canada

## Abstract

In *The Descent of Man*, Darwin speculated that our capacity for musical rhythm reflects basic aspects of brain function broadly shared among animals. Although this remains an appealing idea, it is being challenged by modern cross-species research. This research hints that our capacity to synchronize to a beat, i.e., to move in time with a perceived pulse in a manner that is predictive and flexible across a broad range of tempi, may be shared by only a few other species. Is this really the case? If so, it would have important implications for our understanding of the evolution of human musicality.

## Abstract 2

Are other species able to process basic musical rhythm in the same way that humans do? Darwin supported this intuitive idea, but it is being challenged by new cross-species research.

## Background

Music is a human universal with an ancient history: delicately carved bone flutes made by ice-age hunter-gatherers predate the oldest known cave paintings by several thousand years [1],[2]. While musical forms and meanings vary widely across cultures [3], certain features of human music are widespread [4]. For example, every culture has some form of music with a beat, a perceived periodic pulse that dancers use to guide their movements and performers use to coordinate their actions [5]. Darwin, intrigued by the ubiquity and power of music in human life, felt that our sense of melody and rhythm tapped into ancient and fundamental aspects of brain function, arguing that “The perception, if not the enjoyment, of musical cadences [i.e., melodies] and of rhythm is probably common to all animals, and no doubt depends on the common physiological nature of their nervous systems” [6]. Darwin's intuition seems plausible. Focusing on rhythm, the prevalence of periodic (or near-periodic) rhythms in animal biology (e.g., in heartbeat, gait, and brain activity [7]) makes it reasonable to suspect that beat-based rhythmic processing has ancient evolutionary roots.

Darwin's view suggests that key features of musical beat processing should be similar in humans and other species. For humans, one of the most salient features of musical beat processing is that it links perception and action in an intimate way. We often express our perception of the beat by moving rhythmically (tapping a foot, nodding our head) in time with the beat [8]. That is, humans entrain rhythmic movements to the beat of music, and in social settings (e.g., dancing or marching), this can lead to synchronized rhythmic actions within groups of people [9]. In support of Darwin's view, the ability to entrain actions to a periodic pulse is not uniquely human: several species of frogs and insects are known to call or flash periodically and in synchrony with conspecifics [10]. Indeed, it has been suggested that rhythmic entrainment emerges quite easily in biological systems [11]. A view of rhythmic synchronization as very basic to biological systems informs some current models of musical beat-based processing. For example, in “neural resonance” theory [12],[13], beat perception arises when nonlinear oscillations in the nervous system entrain to (oscillate in synchrony with) external rhythmic stimuli. This theory is in line with Darwin's views because it holds that nonlinear oscillations are ubiquitous in brain dynamics and that the neural entrainment of such oscillations by auditory rhythms is “intrinsic to the physics of the neural systems involved in perceiving, attending, and responding to auditory stimuli” [12].

Such a view is appealing for its generality; yet it faces what biologist Tecumseh Fitch has called “the paradox of rhythm.” As Fitch notes, “Periodicity and entrainment seem to be among the most basic features of living things, yet the human ability (and proclivity) to entrain our motor output to auditory stimuli appears to be very rare.” [14, p. 78]. Stating the paradox more colloquially, Fitch asks “Why don't dogs dance?” Dogs have lived with humans (and our music) for thousands of years, and their brain structure is much more akin to ours than to frogs and insects. Yet they show no spontaneous tendency to synchronize their movements with a musical beat. Indeed, even when humans try to train dogs to dance to music (as in the sport “canine freestyling”), dogs show no evidence of sensing a beat or moving in synchrony with it, unlike their human partners who dance directly beside them [15].

## Challenges to Darwin's View

**Figure 1:**
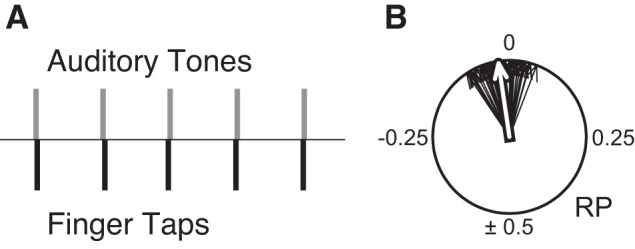
Illustration of how a human adult taps to an auditory metronome. In Figure 1a, the upper gray bars represent the times of five metronome events (brief tones with interonset interval = 600 ms). The lower black bars show tap times, which fall very close to tone onsets. Figure 1b shows summary data for a trial of 40 tones. The relative phase (RP) of each tap is represented by a thin black vector on a unit circle: 0 indicates perfect temporal alignment between taps and tones, negative RP values indicate taps preceding tones, positive RP values indicate taps following tones, and 0.5 indicates taps midway between tones. The white arrow indicates mean relative phase, which is slightly negative in this case (i.e., on average, taps slightly precede tone onsets in time).

Informal observations of dogs aside, more serious challenges to the view that beat-based processing is widespread come from laboratory studies of nonhuman primates [16],[17]. To understand the significance of these studies, it is important to review some key characteristics of how humans synchronize movements to a beat. While humans typically synchronize to the beat of complex auditory stimuli (i.e., real music), basic features of human synchronization to a beat can be studied by having people tap along with a metronome. This is a trivially easy task for most adults, even those with no musical training. Synchronization to a metronome has driven much productive research on sensorimotor processing [18]. Three key features of human synchronization to a metronome are 1) prediction, 2) tempo flexibility, and 3) cross-modality. In terms of the first feature, when humans tap with a metronome they spontaneously align their taps with the beat: taps fall very close to the onset of metronome clicks, typically within a few tens of ms (Figure 1).

This shows that tapping is guided by an accurate *prediction* of when the next beat will occur. In other words, actions are guided by a mental model of time, rather than simply being a reaction to each stimulus (if taps were reactive rather than predictive, they would follow clicks by a few hundred ms). In terms of the second feature, synchronization to a metronome (and to music) in adult humans is very flexible: as long as the interval between beats is between about 300–900 ms (i.e., about 67–200 beats per minute or BPM), humans can achieve synchronization quickly and accurately [19]. This criterion distinguishes human synchronization to a beat from other examples of rhythmic entrainment in nature. Fireflies, for example, can only synchronize to other fireflies in a narrow tempo range around their spontaneous emission rate [20]. In terms of the third feature, humans can synchronize to a beat in a cross-modal fashion; that is, we can easily synchronize by moving silently (e.g., head bobbing), rather than by making sound ourselves (e.g., clapping or vocalizing) [8]. All other species exhibiting synchronous rhythmic behavior do so in the same modality (e.g., frogs calling together, or fireflies flashing together) [21]. While cross-modal synchronization is easy for humans, there is one respect in which our synchronization abilities are modality-biased. When humans synchronize to an auditory metronome, their tapping is much more accurate than when synchronizing with a visual metronome of identical temporal characteristics, a finding that has been replicated in the laboratory for over a century [22]–[24].

Turning to nonhuman primates, if the mechanisms underlying human beat-based processing are widespread in animal brains, one would expect nonhuman primates to show characteristics like those human synchronization exhibits when they are trained to tap to a beat. In the first study to train monkeys (or for that matter, any animal) to tap with a metronome, Hugo Merchant and colleagues obtained surprising results [16]. While the rhesus monkeys (*Macaca mulatta*) could successfully listen to two metronome clicks and then reproduce the same interval by tapping twice on a key, they had great difficulty learning to tap in synchrony with a metronome of several beats. Specifically, each monkey took over a year of training to learn the metronome task, and when tested, their taps were always a few hundred ms after each metronome click rather than aligned with it. This suggests that their behavior was dominated by reaction rather than anticipation (although they did react more quickly to metronome events than to randomly timed events, thus showing some modest anticipation abilities). The monkeys learned to tap with metronomes at several different tempi, but spontaneous tempo flexibility was not tested (i.e., training at one tempo and testing at another tempo). Finally, unlike humans, the monkeys showed similar tapping variability for auditory and visual metronomes.

Thus it seems that human-like beat-based processing may not come easily to monkeys. Surprisingly, these differences may extend from synchronization to pure perception of a beat (in the absence of movement). This is suggested by subsequent research in the Merchant lab, which used neural measures to examine beat perception in monkeys who were sitting still. In this work, modeled on previous work with humans [25], monkeys were presented with a repeating auditory rhythmic pattern (which was more complex than a metronome but which had an underlying beat) while EEG data were collected. Unlike humans tested with these stimuli, the monkeys did not show a neural correlate of beat perception [26].

Naturally one wonders if similar results would have been obtained with great apes, who are much more closely related to humans, and who are known to drum in the wild [27]. While no studies have examined neural responses to a beat in apes, the first study of synchronization to an auditory metronome in great apes was recently published. In this study, three chimpanzees (*Pan troglodytes*) were trained to tap rhythmically on a keyboard and were tested for spontaneous synchronization to a metronome at three different tempi [17]. One chimp synchronized her taps to the metronome at one tempo but not at the other two tempi, while the other chimps did not synchronize at any tempi. Thus while chimps may have the capacity for anticipatory synchronization (not yet evident in monkeys), so far they show no evidence of significant tempo flexibility.

What are we to make of these challenges to Darwin's view of animal rhythmic processing? One possibility is that different training and testing methods would produce different results, and that human-like synchronization to a metronome is possible in monkeys and apes. Indeed, given how few studies have examined synchronization to an auditory beat in nonhuman primates, this possibility deserves to be explored. For example, future studies with monkeys could use reaching tasks (at which monkeys are known to be adept [28]) and a touch screen. Specifically, two illuminated circles could appear periodically and in alternation at fixed positions on the left and right side of the screen, and the monkey could be trained to use one hand to touch each circle before it disappears. This would require anticipatory (rather than reactive) reaching movements, in order to touch each circle on the screen while it was illuminated. A tone could be played at the same time as each circle is displayed, and once the task was learned, the visual stimulus could be faded-out to make the stimulus auditory only. Once the task was mastered at one tempo, generalization to other tempi could be tested.

## Could Beat-Based Processing Be Species-Restricted?

Might it be that only a few species have the capacity to synchronize rhythmic movements to a beat in a manner similar to humans? In theoretical writings that predated the recent work on synchronization in nonhuman primates, I suggested that this might be the case [29]. Specifically, I proposed the “vocal learning and rhythmic synchronization hypothesis” (henceforth, “vocal learning hypothesis”), which suggests that the capacity to synchronize with a musical beat resulted from changes in brain structure driven by the evolution of complex vocal learning. Complex vocal learning is learning to produce complex vocal signals based on auditory experience and sensory feedback. This is a rare trait in nature: most animals (including all nonhuman primates) have a small set of instinctive vocalizations which they can modify in only modest ways in terms of their acoustic patterning. Vocal learning occurs in just three groups of birds (songbirds, hummingbirds, and parrots) and a few groups of mammals, including humans, elephants, and some cetaceans, seals, and bats [30]–[35]. The neurobiology of vocal learning has been best studied in birds, where the brain structure of vocal learners has been compared in great detail to that of vocal nonlearners (such as chickens or pigeons). This work has revealed that vocal learning is associated with specialized neural circuitry, including specializations in forebrain premotor areas, the basal ganglia, and their connections [36]. One motivation for the vocal learning hypothesis was that human neuroimaging revealed that premotor and basal ganglia regions are important for beat-based processing. Indeed, neuroimaging reveals that pure beat perception (even in the absence of overt movement) engages mid-to-dorsal premotor regions and basal ganglia regions (e.g., the putamen) [37],[38], which become functionally coupled to auditory regions [39]. It has been theorized that this functional coupling plays a role in our ability to predict the timing of beats [40],[41], a key feature of beat-based processing. More generally, moving in synchronization with a beat requires tight auditory-motor coupling in the service of an auditory model (a mental model of a temporal interval), just as vocal learning requires tight auditory-motor coupling in the service of an auditory model (the sound an animal is trying to imitate).

Of course, even if vocal learning and synchronization to a beat both engage premotor-basal ganglia-auditory networks, it may seem puzzling to claim that the two abilities are related, since they use different parts of the motor system (the vocal tract vs. the limbs, trunk, head, etc.). Thus the vocal learning hypothesis entails the idea that the evolution of vocal learning led to more general integration of auditory and motor regions of the brain than just the circuits connecting auditory and vocal motor control centers [42].

**Figure 2:**
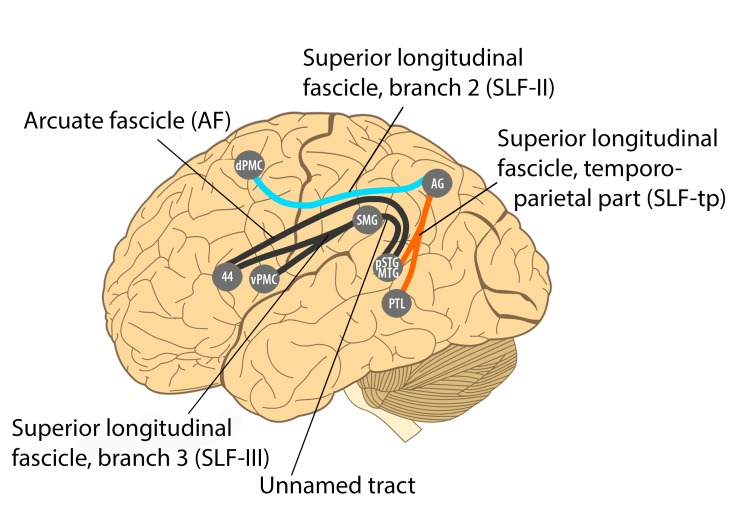
Recent summary diagram of long-distance fiber tracts in the human dorsal auditory stream (adapted, with permission, from [43]). Of particular interest here are connections between auditory regions in the posterior superior temporal gyrus/middle temporal gyrus (pSTG/MTG) and the angular gyrus (AG) of the parietal cortex, and connections between the angular gyrus and the dorsal premotor cortex (dPMC). These connections correspond to two branches of the superior longitudinal fasciculus (SLF): the SLF temporo-parietal branch (SLF-tp) and the 2^nd^ branch (SLF II). Interestingly, both tracts appear to play a role in the human ability to repeat what is heard [43], a key part of vocal learning. PTL: posterior temporal lobe; SMG: supramarginal gyrus; vPMC: ventral premotor cortex; 44: Brodmann area 44 (part of Broca's area).

Of particular interest in this regard are connections in the human brain between auditory superior temporal cortical regions and dorsal premotor regions of the frontal cortex, via the parietal cortex [43] (Figure 2, orange line connecting posterior superior temporal gyrus/middle temporal gyrus [pSTG/MTG] with angular gyrus [AG], and light blue line connecting angular gyrus with dorsal premotor cortex [dPMC]). As shown in Figure 2, these connections correspond to two branches of a large neural fiber pathway known as the superior longitudinal fasciculus (SLF): specifically, the temporo-parietal part (SLF-tp) and branch 2 of the SLF [SLF II].

These connections are part of a “dorsal auditory stream” linking auditory and premotor regions, which is thought to play a role in sensorimotor transformations in speech and other domains [44]. Importantly, this pathway (especially the part connecting the auditory and superior parietal cortex, i.e., SLF-tp) may be much more developed in humans than in nonhuman primates [45],[46], which could account for differences between humans and other primates in the ability to synchronize to a beat (see [41] for an extended treatment). This issue merits further comparative study.

One virtue of the vocal learning hypothesis is that it makes testable predictions about what kinds of animals can vs. cannot synchronize to a beat in a human-like way. Specifically, it posits that vocal nonlearners lack this capacity, a prediction that has so far been borne out in primate research (though more work is needed, as noted above). In contrast, it predicts that vocal learners may have this capacity. (The qualification of “may” is important, because the hypothesis says that neural circuitry related to vocal learning is necessary for human-like synchronization, but does not claim that it is sufficient [15],[47],[48].) Support for the hypothesis comes from studies showing that several species of parrots can synchronize to the beat of music in a manner that is predictive, tempo-flexible, and cross-modal [49]–[51]. In two of these studies [49],[50], the parrots (who were human pets) appear to have developed this behavior without any formalized training, perhaps by observing humans (though they can now synchronize to music without a human model). It should be noted, though, that parrots do not synchronize to a beat as well as adult humans, and show transient “bouts” of synchronization to a beat, perhaps akin to human children [52]. Thus further work is needed to directly compare the synchronization abilities of parrots and nonhuman primates. In doing this work, it will be important to document whether synchronization to a beat emerges spontaneously, as it does in humans (i.e., via exposure to beat-based rhythms and to visual models of others synchronizing), or if it requires explicit reinforcement training. This is important because these two different ways of acquiring synchronization abilities may reflect differences in the underlying mechanisms.

On a related note, an important question for future work is whether the behavioral similarities in synchronization to a beat in parrots and humans are due to similar underlying neural mechanisms, or if these similarities are superficial and rely on rather different neural circuits (Box 1). The vocal learning hypothesis takes the former view. Since parrots are not known to synchronize to a beat as part of their natural behavior in the wild, the hypothesis implies that this capacity emerges as a serendipitous byproduct of brain circuitry that evolved for other reasons, i.e., for vocal learning.

**Box 1. Parrot and human synchronization to a beat: similar or distinct brain mechanisms?**

Research with parrots has provided the first experimental evidence that nonhuman species can synchronize movements to a beat in a human-like fashion [49]–[51] (for video examples, see http://www.youtube.com/watch?v=ERpIWTh18cY). Is this behavioral similarity simply a superficial resemblance, resting on rather different brain mechanisms in parrots and humans? Similarity of behavior is no guarantee of similar underlying mechanisms. For example, a parrot can say “Polly want a cracker,” but this emulation of speech is produced by very different articulatory mechanisms than those used in human speech [79]. On the other hand, similar behavior in distantly related species can be supported by similar mechanisms. Vocal learning, for example, appears to have arisen independently in three distantly related groups of birds (parrots, songbirds, and hummingbirds). Yet a broadly similar set of brain nuclei appears to be involved in each case, pointing to “deep homology,” i.e., the convergent evolution of a trait based on similar biological mechanisms, possibly due to underlying genetic constraints on how those traits can be assembled [80],[81]. Could vocal learning in birds and mammals also be a case of deep homology, involving broadly similar premotor-basal ganglia-thalamic neural circuits? The biologist Tecumseh Fitch has argued for this view [81]. One fact that makes this argument interesting is that a gene important for motor control of human speech, *FoxP2*, is also expressed in avian brain regions important for motor control of learned song [81]–[83]. This is consistent with the idea that vocal learning in birds and humans has a similar underlying biology [84]. If this is the case, and if the capacity for synchronization to a beat is related to vocal learning circuitry, this would support a deep homology between the brain mechanisms used in synchronization to a beat in vocal learning birds and humans.

## Possible Support for Darwin's View

Thus far I have discussed two very different views of beat-based processing: either as reflecting ancient and widespread aspects of brain function or as the result of specialized brain networks that exist in a small subset of animal species. If the former view is correct, then many animal species, if given the right training, should exhibit the capacity for beat-based processing.

Recently the capacity to synchronize to a musical beat has been demonstrated in a California sea lion (*Zalophus californianus*) [53]. Like parrots, sea lions are not known to synchronize movements to rhythmic sounds in the wild. Yet the sea lion learned to synchronize silent head bobs with an auditory beat (although this required structured reinforcement training, unlike with parrots). Crucially, the sea lion showed tempo flexibility: after training to synchronize at one tempo, she could generalize this behavior to novel tempi. This is potentially strong evidence in favor of Darwin's view and against the vocal learning hypothesis, since this species is not known to be a vocal learner. However, sea lions (family Otariidae) are related to true seals (family Phocidae) and to walruses (family Odobenidae), which are known vocal learners [30],[34],[54]. Hence the absence of evidence for vocal learning in sea lions is not strong evidence of absence of this capacity or its underlying neural mechanisms. To test the prevailing view that sea lions are much less vocally flexible than seals, behavioral training studies of vocal flexibility in sea lions are needed, particularly since the most recent experimental studies of sea lion vocal flexibility date from the 1960s and 1970s [54]. Neural studies would also be of interest, e.g., structural neuroimaging of sea lions vs. seal brains using diffusion tensor imaging (DTI), a type of magnetic resonance imaging (MRI) that can visualize white matter pathways in living brains. DTI could be used to search sea lion brains for neural connections associated with vocal learning and for other connections potentially relevant for beat processing (e.g., the temporo-parietal branch and 2^nd^ branch of the superior longitudinal fasciculus, Figure 2). It may be, for example, that sea lions retain auditory-motor circuits inherited from a vocal-learning common ancestor of seals, sea lions, and walruses [55], even though they do not show obvious signs of vocal learning in captivity. (Flexible auditory-motor integration may be useful to sea lions because of their amphibious lifestyle: they produce and perceive a diverse set of vocalizations in two very different environments, i.e., above and underwater [56]–[58].) If future work shows that sea lions have very limited vocal flexibility and lack the neural circuitry associated with vocal learning, this would seriously challenge the vocal learning hypothesis. It would however leave open the broader question of whether the ability to synchronize to an auditory beat in a human-like way is species-restricted, and if so, why only certain animals have this capacity.

## Where Do We Go from Here?

The range of species capable of human-like synchronization to a beat is currently an unsolved mystery. Apart from further research on parrots and nonhuman primates, which other animals should be tested for this ability? In terms of vocal learners, further work is needed to find out whether the capacity to synchronize to a beat is latent in all vocal learners (e.g., including bats), or only in a subset of vocal learners who also have other key traits. Parrots, for example, can imitate nonvocal gestures and are also deeply social creatures who may have a propensity for coordinated movement with social partners [59]. It may be that these other traits are necessary, in addition to vocal learning, to create the capacity for human-like synchronization to a beat [15],[47],[48]. If this is the case, then only vocal learners with these other traits, such as dolphins [60], may be able to synchronize to a beat in a human-like fashion.

In terms of vocal nonlearners, one animal of particular interest is the domestic horse (*Equus ferus caballus*), a vocal nonlearning animal that (unlike sea lions) has no close vocal-learning relatives. In favor of Darwin's views on musical rhythm, there are anecdotal accounts of horses spontaneously synchronizing their gait to the beat of music, even when they have no rider (who could unintentionally give them cues to the beat). This makes them an ideal test case for Darwin's view, since the vocal learning hypothesis predicts that they lack human-like capacities for synchronizing to a musical beat. Using new methods for testing synchronization to music in horses ([61]–[63], Movie S1), this prediction can now be tested.

Stepping back to a larger view, studies of beat-based processing in other animals are part of a small but growing body of cross-species research on music processing (e.g., [16],[17],[26],[49],[50],[64]–[78]). Such research is in its infancy, but is worth pursuing because it provides an empirical approach to studying the evolutionary history of human musicality. Specifically, it can help identify which aspects of our nonlinguistic auditory processing are broadly shared with other species, which aspects are shared with just a few other species, and which are uniquely human. It is important to note that such work is essentially Darwinian in its approach. That is, even if Darwin was wrong about the widespread nature of musical rhythm processing, the cross-species approach to evolutionary studies that he championed will undoubtedly lead us to a deeper understanding of the biological roots of human music.

## Supporting Information

Movie S1 **Illustration of a new method for testing if horses synchronize their gait to the beat of music, from** [**61**] **.** In this “circular trotting to music” method, a horse trots in circles around a trainer while ambient music with a clear beat is played in the arena. The trainer wears closed-ear headphones and listens to masking music with no beat (e.g., meditation music), in order to avoid giving the horse inadvertent cues to the musical beat. Using frame-by-frame video analysis and quantitative statistical methods, the timing of the horse's footfalls are compared to the timing of musical beats to test for synchronization. The test is repeated at several different tempi to examine tempo flexibility, as in [49].
(MP4)
