# Correction: The Evolutionary Biology of Musical Rhythm: Was Darwin Wrong?

**DOI:** 10.1371/journal.pbio.1001873

**Published:** 2014-05-07

**Authors:** The PLOS Biology Staff

## Notice of Republication

This article was republished on April 22, 2014, to correct an error in Figure 2 that was introduced during the production process. Figure 2 was missing information in the online version of the article. The figure in the original PDF version of the article is correct.
